# Multiple pathogens co-exposure and associated risk factors among cattle reared in a wildlife-livestock interface area in Kenya

**DOI:** 10.3389/fvets.2024.1415423

**Published:** 2024-07-25

**Authors:** Sophina Manyenya, Daniel Nthiwa, Harrison Osundwa Lutta, Mathew Muturi, Richard Nyamota, Athman Mwatondo, Grace Watene, James Akoko, Bernard Bett

**Affiliations:** ^1^Department of Biological Sciences, University of Embu, Embu, Kenya; ^2^International Livestock Research Institute, Nairobi, Kenya; ^3^Biotechnology Research Institute, Kabete Centre, Kenya Agricultural and Livestock Research Organization, Nairobi, Kenya; ^4^Kenya Zoonotic Disease Unit, Ministry of Health and Ministry of Agriculture, Livestock, and Fisheries, Nairobi, Kenya

**Keywords:** foot-and-mouth disease virus, *Brucella* spp., *Leptospira* spp., *Coxiella burnetii*, co-exposure, livestock-wildlife interface, cattle, Kenya

## Abstract

**Introduction:**

Understanding multi-pathogen infections/exposures in livestock is critical to inform prevention and control measures against infectious diseases. We investigated the co-exposure of foot-and-mouth disease virus (FMDV), *Brucella* spp., *Leptospira* spp., and *Coxiella burnetii* in cattle in three zones stratified by land use change and with different wildlife-livestock interactions in Narok county, Kenya. We also assessed potential risk factors associated with the transmission of these pathogens in cattle.

**Methods:**

We identified five villages purposively, two each for areas with intensive (zone 1) and moderate wildlife-livestock interactions (zone 2) and one for locations with low wildlife-livestock interactions (zone 3). We sampled 1,170 cattle from 390 herds through a cross-sectional study and tested the serum samples for antibodies against the focal pathogens using enzyme-linked immunosorbent assay (ELISA) kits. A questionnaire was administered to gather epidemiological data on the putative risk factors associated with cattle’s exposure to the investigated pathogens. Data were analyzed using the Bayesian hierarchical models with herd number as a random effect to adjust for the within-herd clustering of the various co-exposures among cattle.

**Results:**

Overall, 88.0% (95% CI: 85.0–90.5) of the cattle tested positive for at least one of the targeted pathogens, while 41.7% (95% CI: 37.7–45.8) were seropositive to at least two pathogens. FMDV and *Brucella* spp. had the highest co-exposure at 33.7% (95% CI: 30.9–36.5), followed by FMDV and *Leptospira* spp. (21.8%, 95% CI: 19.5–24.4), *Leptospira* spp. and *Brucella* spp. (8.8%, 95% CI: 7.2–10.6), FMDV and *C. burnetii* (1.5%, 95% CI: 0.7–2.8), *Brucella* spp. and *C. burnetii* (1.0%, 95% CI: 0.3–2.2), and lowest for *Leptospira* spp. and *C. burnetii* (0.3%, 95% CI: 0.0–1.2). Cattle with FMDV and *Brucella* spp., and *Brucella* spp. and *Leptospira* spp. co-exposures and those simultaneously exposed to FMDV, *Brucella* spp. and *Leptospira* spp. were significantly higher in zone 1 than in zones 2 and 3. However, FMDV and *Leptospira* spp. co-exposure was higher in zones 1 and 2 than zone 3.

**Discussion/conclusion:**

We recommend the establishment of a One Health surveillance system in the study area to reduce the morbidity of the targeted zoonotic pathogens in cattle and the risks of transmission to humans.

## 1 Introduction

Livestock production is a significant economic activity that employs about 1.3 billion people worldwide (1). In Kenya, livestock production contributes approximately 42% to the agricultural gross domestic product (GDP) and about 12% of the national GDP (2). While livestock production is carried out across Kenya, the arid and semi-arid lands (ASALs) that are also inhabited by wildlife provide good rangelands for livestock farming. An example is the Maasai Mara ecosystem (MME) in Narok County including the Maasai Mara National Reserve (MMNR) and bordering areas. Livestock are critical resources in these areas as they contribute directly to the households’ income and food security (3). The local inhabitants in these locations also obtain environmental products such as firewood, medicinal plants, wild foods, and water for livestock and domestic use. Communities living near the MMNR boundary extract more of these environmental benefits than those in far-off areas (4).

The existence of the MME is facing a myriad of challenges including the unprecedented land use shifts attributed to high population growth, land privatization, climate change, and urbanization (5, 6). These anthropogenic influences have also been linked with the significant reduction of wild ungulate species populations in the area (7). The emerging land use transitions in the area include the creation of wildlife conservancies in private and communal lands near MMNR to conserve wildlife and foster tourism and also generate income for the local communities and revenues for the country (8). These conservancies also have well established management arrangements that allow the local inhabitants to graze their livestock in these environments. As previously reported (9), other land use modifications in MME are shown by the change from the semi-nomadic livestock farming practice in the areas around MMNR to sedentary or pure/mixed crop-livestock farming in lands distant from MMNR. Although posited as a sustainable approach that allows livestock and wildlife co-existence, the new land use strategies present unique challenges to farmers and their livestock in this area. The challenges reported in the area include the competition for ecological resources between livestock and wildlife, crop destruction, livestock predation, and human injuries or death due to attack by wildlife (10). Cross and/or within species transmission of infectious agents can also occur indirectly in these environments through contaminated surface water, fomite and forage or as a consequence of intensified effective contact rates between infected and susceptible hosts (11). For instance, earlier studies in the area have documented zoonotic pathogens such as anthrax (12), *Leptospira* spp. and *Brucella* spp. in livestock (13). These pathogens also infect diverse wildlife including the African buffalo (*Syncerus caffer*), notable species in the study area, that can modify the transmission patterns of the considered pathogens.

Most of the past epidemiological studies implemented in MME among livestock populations concentrated more on single pathogen infections or exposures (9, 14, 15) or had a very narrow focus on co-exposure (13). Therefore, investigations on the simultaneous infections and/or exposure of livestock to multiple infectious agents are limited in the study area. We used FMDV, *Brucella* spp., *Leptospira* spp., and *C. burnetii* as pathogens of interest to understand their co-exposure and identify associated risk factors among cattle kept in three confluent zones stratified by land use types and with low to high wildlife-livestock interactions. Compared to the other study pathogens, there is also limited data on the burden, distribution and epidemiology of *C. burnetii* in livestock in the area. FMDV within the genus *Aphthovirus* causes foot-and-mouth disease (FMD), a transboundary viral disease of the cloven-hoofed domestic and wild animals (16). *Brucella* spp., *Leptospira* spp., and *C. burnetii* are globally spread bacterial zoonotic pathogens that cause brucellosis, leptospirosis and coxiellosis or Q fever, respectively, in diverse hosts including livestock, wildlife and humans (17–19). In Kenya, these zoonotic agents cause high morbidities in both livestock and humans (20), and extensive economic consequences (21). All the targeted pathogens also cause multiple common reproductive disorders in infected livestock such as abortions, reproductive failures, stillbirths and weak offspring, besides case fatalities (17, 19, 22, 23). The findings of this study will inform the development of integrated prevention and control strategies for these pathogens in the area including the establishment of an active biosurveillance system. Our results also shed more light on the ecology and epidemiology of the investigated pathogens in a livestock-wildlife interaction area.

## 2 Materials and methods

### 2.1 Study area

**Figure 1:**
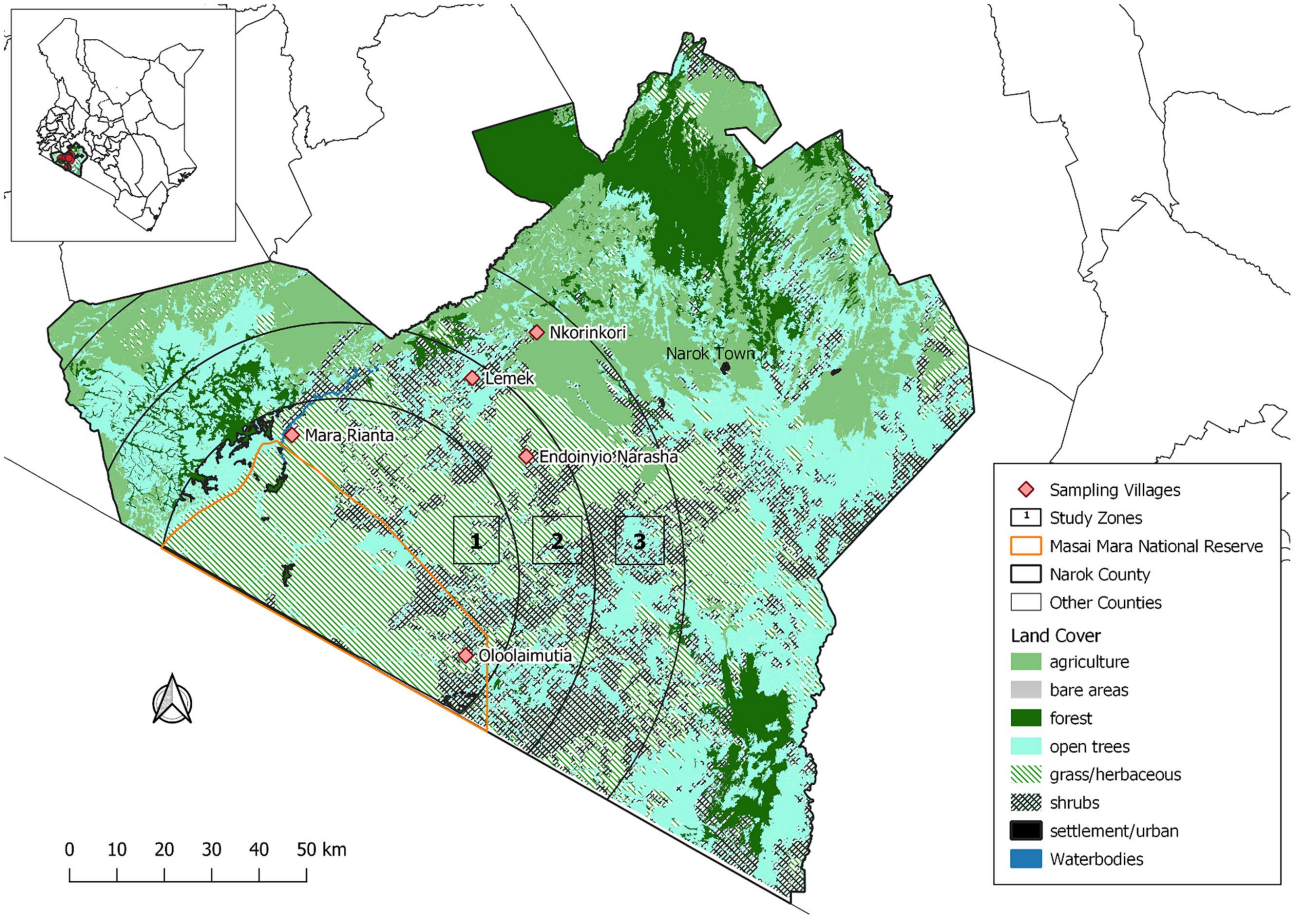
Map showing sampling zones within the Maasai Mara ecosystem.

We implemented this study in the MME within Narok County in Kenya (Figure 1). The study area has been previously described (9). The MME, an area of about 6,000 km^2^, includes the MMNR and the surrounding areas. The MMNR (1,530 km^2^) is a protected area that is continuous with the Serengeti National Park in Tanzania. The study area receives bimodal rainfall ranging from 500 to 1,300 mm annually (5). From the MMNR boundary, the study area was stratified into three contiguous zones with changing land use types. Zone 1 was located 20 km from MMNR and represented areas with high wildlife-livestock interface, while zone 2 (between 20 and 40 km from MMNR) were the areas with moderate wildlife-livestock interactions. Areas more than 40 km from MMNR represented zone 3 with low wildlife-livestock interactions. In zone 1, cattle are mainly grazed illegally in the MMNR and surrounding wildlife conservancies in semi-nomadic pastoral systems, while in zone 2, they are pastured in fenced farms in sedentary husbandry systems although the entire area is not fenced and livestock still interact with wildlife. Crop cultivation, mainly maize and wheat, and/or livestock production are carried out in zone 3. Five representative villages, two each for zones 1 and 2 and one for zones 3, with comparable characteristics to those of the above-explained zones were purposively selected for sampling.

### 2.2 Study design and sample size estimation

Cattle sampling was carried out between September 2016 and July 2017 through a cross-sectional study as previously reported (9, 13). Briefly, the calculation of sample size (*n*) was done using the formula, $n=\mathrm{Deff}\times \frac{1.96^{2}\times P\times \left(1-P\right)}{d^{2}}$where *Deff* denotes the design effect, *d* is the acceptable standard error of 5%, and p is the expected seroprevalence for each targeted pathogen in the cattle population in the area (24). As mentioned in the introduction, there are studies on single pathogens done in the same area and different seroprevalence estimates for some of these pathogens exists. Nevertheless, in our study, the expected apparent seroprevalence for each target pathogen was assumed to be 50% to generate maximum possible sample size. The initially calculated sample size of 384 cattle based on the above parameters was corrected for design effect since in our sampling scheme, households and cattle were primary and secondary sampling units, respectively. We calculated the design effect using the formula *deff* = 1 + *ICC*(b–1), where *ICC* denoted the intra-cluster correlation coefficient and *b* the number of cattle sampled per herd (24). We used an ICC estimate of 0.1 for all pathogens as informed by a comparable study implemented in a pastoral area (25). Blood was collected from a random sample of three cattle in each herd. The computed design effect was 1.2 and it gave a corrected target sample size of 465 cattle from 155 (465/3) herds in each zone. Nevertheless, we sampled a total of 1,170 cattle from 390 herds in the three zones, allocated proportionately between zones based on the number of herds. The distribution of sampled animals by zones were as follows; 465 animals from 155 herds, each for zones 1 and 2, and 240 animals from 80 herds in zone 3.

### 2.3 Household selection, animal sampling, and sample processing

Before animal sampling, we compiled a list of cattle-keeping households for each selected village assisted by the respective area chiefs. Simple random sampling was then applied to select households (representing cattle herds) for sampling. Only cattle aged 1 year and above were sampled as these animals regularly interact with wild animals and/or livestock from other herds at shared resources such as watering points or grazing fields compared to calves that are grazed on pastures within household surroundings. Given this, we assumed that cattle aged ≥1 year had a higher probability of exposure or infections with the investigated pathogens than calves. Up to 10 mL of blood was drawn into plain barcoded vacutainer tubes from the jugular vein of each animal. The samples were carried in cool boxes filled with dry ice to the Kenya Wildlife Service (KWS) laboratory within MMNR for processing on the same day of collection. The blood samples were centrifuged for 6 min at 5,000 revolutions per minute (rpm) and obtained serum aliquoted into two barcoded cryovials. These samples were transported in a portable freezer to the International Livestock Research Institute (ILRI), Nairobi, where they were stored at −20°C in the Biosciences laboratory facility before being tested for immunoglobulins against the targeted pathogens.

### 2.4 Data collection

During cattle sampling, a questionnaire was administered in each selected household to gather epidemiological information on potential animal and herd level risk factors for the transmission of the study pathogens in cattle. This information included animal sex, age, herd size, sampling sites (villages and zones), herd management practice, source of breeding bulls, history of abortions in sampled herds in the past year, purchase of livestock in the past year, cattle contact with others from a different herd at watering points or during grazing; whether cattle shared watering points within or between villages, and grazing strategies such as cattle utilizing a common grazing reserve, grazing of cattle in the MMNR, on pastures shared within villages or those shared between villages. In addition, we also collected data on whether livestock interacted with wild animals and the wildlife species they interacted with in each study location.

### 2.5 Testing of serum samples

All the 1,170 serum samples were tested using commercial immunological assays to detect antibodies against FMDV, *Brucella* spp., and *Leptospira* spp. as earlier described (9, 13). The testing of samples for specific antibodies to these three pathogens was done between 2017 and 2018. Due to logistical challenges, only 589 (50.3%, *n* = 1,170) randomly selected samples and approximately proportionate to the number of cattle sampled per zone were screened for antibodies against *C. burnetii* later in 2023. Briefly, the samples were tested for antibodies against FMDV non-structural proteins (NSPs) using two anti-NSPs based ELISA kits to differentiate convalescent animals from vaccinated. Specifically, the samples were tested using PrioCHECK FMDV NS blocking ELISA (Prionics, AG, Netherlands) and FMDV 3ABC-trapping ready-to-use kits [Istituto Zooprofilattico Sperimentale della Lombardia edell’Emilia Romagna (IZSLER), Italy] as per manufacturer’s guidelines. Animals were categorized as seropositive based on the parallel interpretation of the two anti-NSPs results. The samples were screened for immunoglobulins (IgG1) against *Brucella abortus* using PrioCHECK *Brucella* Antibody 2.0 indirect ELISA kit, while the testing of *Leptospira interrogans* serovar *hardjo* antibodies was carried out using PrioCHECK *Leptospira hardjo* indirect ELISA, all from Prionics AG, Netherlands. Tested animals were classified as either seropositive or seronegative for the above pathogens based on the manufacturers’ cut-off values of the respective ELISA kits for each pathogen.

The screening of samples for IgG antibodies against *C. burnetii* was conducted using an indirect serological kit (IDEXX laboratories, Westbrook ME, United States), as per the manufacturer’s instructions. In each 96 well test plate, the serum samples together with the positive and negative control sera were tested in duplicates. The optical densities (ODs) recorded at 450 nm for all wells were used to compute percentage positivity (PP) ratio for each tested serum as follows; mean sample OD – mean OD of negative control divided by mean positive control OD - mean OD of negative control multiplied by 100%. According to manufacturer’s recommendations, cattle were considered as positive, borderline (suspect) and negative if the PP was more than 40%, between 30–40%, and <30%, respectively. Repeated testing of samples with borderline results was conducted.

### 2.6 Data analyses

#### 2.6.1 Descriptive analyses

Prior to data analyses, we merged laboratory and questionnaire epidemiological metadata into a single file in the R software environment, version 4.1.3 (26). The dependent variables of interest were based on the various possible combinations of the selected pathogens. We categorized animals that tested positive to any two target pathogens as having co-exposure while those with antibodies against more than two pathogens were considered to have multiple pathogen exposure. The preliminary descriptive results computed were the overall seroprevalence estimates for the above-mentioned outcome variables. Cross-classification tables with *χ*^2^-test being incorporated were created using the *CrossTable* command in the *gmodels* package (27) to generate these results and assess the crude associations between the various outcomes and categorical factors. The *epi.conf* function in the *epiR* package (28) was then used to estimate adjusted 95% confidence intervals for seropositivity estimates due to the design effect given the cluster sampling scheme. Cattle herd size being a quantitative variable was first checked using the Shapiro–Wilk test to determine if the residuals were normally distributed before further analyses.

#### 2.6.2 Statistical modeling

**Figure 2:**
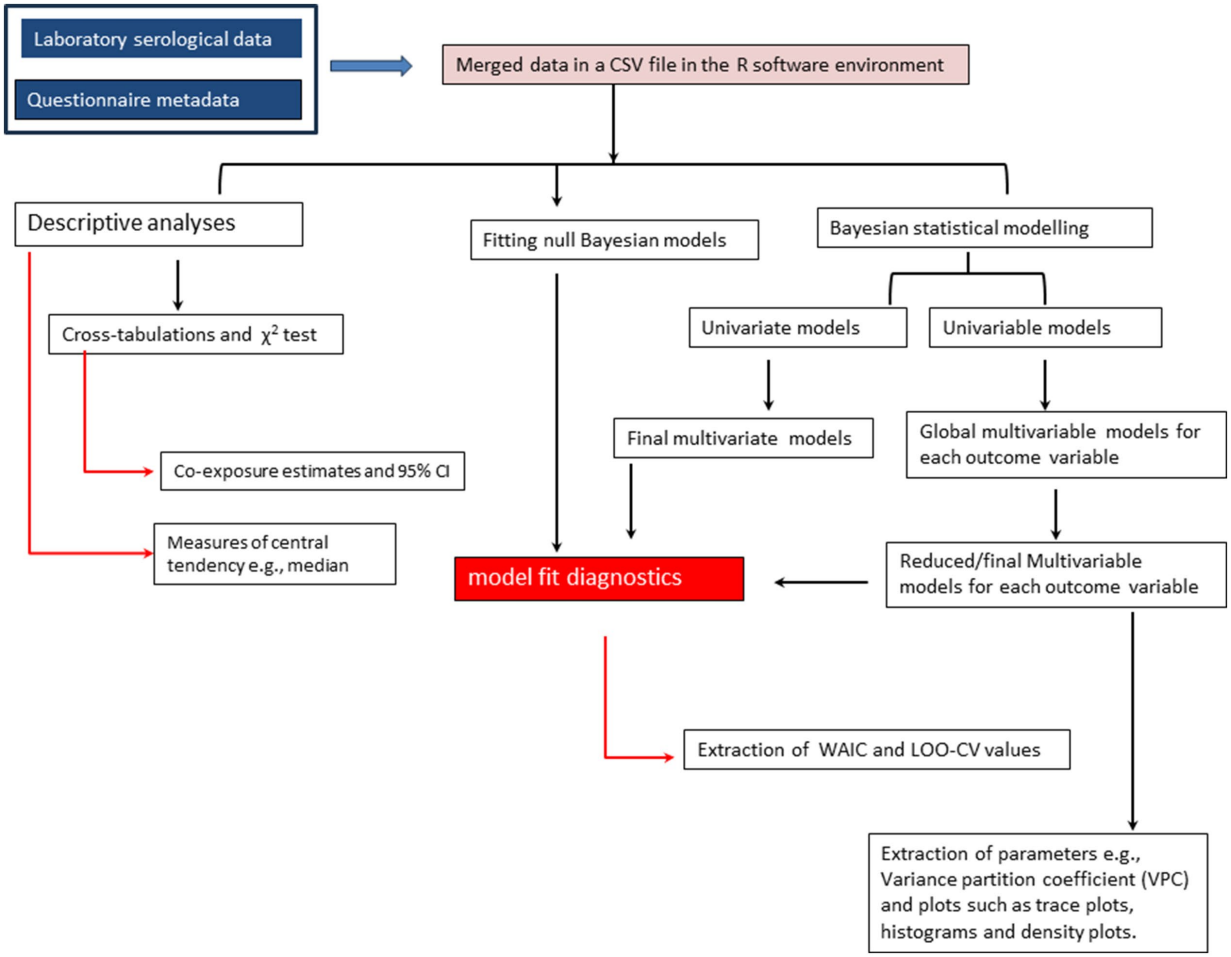
A flowchart showing the steps followed during data analysis.

Risk factor analyses were conducted using Bayesian hierarchical models that are more flexible and robust than the classical “frequentist” methods as they permit inclusion of multiple response variables and prior information on the distribution of the parameters (24). Although these modeling approaches also differ in many other aspects including how the model parameters are estimated (24), Bayesian statistical approaches are useful when maximum likelihood estimation procedures in the classical methods reach their limits and fail to generate model outputs as was the case in our study. While fitting the Bayesian models, only six dependent variables with overall seropositivity estimates of ≥1.0 were considered. Outcome variables including the exposure of cattle to at least one, two and three pathogens were excluded in the analyses as we aimed to identify potential risk factors associated with the seropositivity of specific co- or multiple pathogens. In our analyses, we first fitted two exploratory univariate models for outcome variables based on sample sizes of 589 and 1,170, respectively, to allow simultaneous predictions of parameters. These models were fitted with an auto-correlated specification for the dependent variables. We further carried out univariable Bayesian analyses to assess the unconditional associations between the selected outcomes and independent variables. From both univariate and univariable Bayesian models, variables with mean posterior distributions above zero and 95% credible intervals without a zero were considered significant (24). Statistically significant variables in the univariate analyses were selected to fit global multivariate models following the same procedure described for the univariate models above, while those significant for each outcome of interest by the univariable models were used to fit respective multivariable Bayesian models. All the Bayesian models were implemented with a varying effect for herd ID using the *mvbind* function in *brms* package (29), an interface to *stan* probabilistic programming language (C++) (http://mc-stan.org/) for implementing full Bayesian inference (30). While fitting data to these models, each dependent variable being a Bernoulli random variable *Y* with binary outcomes drawn from {0,1}, where 0 denoted failure (seronegative) and 1 success (seropositive) was defined by the probability density function; $fY\left(y\right)=p^{y}{\left(1-p\right)}^{1-y}$ (31). The logit link function was also specified in the models. The models had four chains, each with 2,000 iterations, 1,000 warms up, and 4,000 post-warm up draws. Due to the variability of seroprevalence estimates of the investigated pathogens, we used standard non-informative flat and vectorized student’s T distributed priors centered on zero for fixed and varying effects, respectively, all default in the *brms* package. The Hamiltonian Monte Carlo and No-U-Turn (NUTS) sampling algorithms were used to estimate the posterior distributions of the parameters (32). These algorithms converge rapidly, especially for complex hierarchical models, relative to Markov-Chain Monte-Carlo (MCMC) (33). The final multivariable Bayesian models were derived through backward stepwise deletion method. Initially, global models were fitted for each dependent variable using significant variables from the respective univariable Bayesian analyses. Non-significant correlates were then removed through single stepwise deletions to obtain final reduced models. The variance partition coefficient (VPC), a variance ratio comparable to ICC in the classical statistical methods, was obtained from the simulated random effect variance of the posterior predicted distribution of each final model using the *variance*_*decomposition* function in *performance* package (34). The final models were also evaluated for the convergence of the algorithms by checking the Rhat diagnostic values and trace plots. The adequacy of each final model was checked graphically using posterior predictive checks implemented using the *pp_check* function in *brms* package. Lastly, we fitted null intercept-only models for each selected outcome and compared these models with the corresponding global and reduced competing models using Watanabe Akaike Information Criterion (WAIC) and leave-one-out-cross-validation (LOO-CV) approach. A flowchart summarizing the above statistical analyses steps is presented as Figure 2.

## 3 Results

### 3.1 Descriptive results

A total of 920 (78.6%) female and 250 (21.4%) male animals were sampled from 390 cattle herds. All the sampled cattle herds were indicated to interact with wildlife. Although the diversity of wildlife species that interact with livestock was indicated to decrease with distance from the areas bordering the MMNR (zone 1), the African buffalo (*Syncerus caffer*), Grevy’s Zebra (*Equus grevyi*), elephants (*Loxodonta africana*), blue wildebeests (*Connochaetes taurinus*), giraffe (*Giraffa* spp.) and impala (*Aepyceros melampus*) were noted as examples of common species in the high interface area, while in the moderate and low interface areas, Grevy’s Zebra and blue wildebeests are prevalent. The overall median cattle herd size was 50 (range; 4–570). The median cattle herd sizes disaggregated by zones were 70 (5–570), 50 (4–300) and 45 (6–300) for zones 1, 2 and 3, respectively, while based on the livestock production system, these estimates were 49 (4–300) and 70 (5–570) for sedentary and pastoral systems, respectively.

The overall seropositivity estimates of the various co- or multiple pathogen exposures in cattle and their distributions by zones are given in Table 1. Overall, 88.0% (95% CI: 85.0–90.5) of the cattle were seropositive for at least one of the targeted pathogens, while 41.7% (95% CI: 37.7–45.8) were exposed to at least two pathogens. The highest co-exposures were observed between FMDV, *Brucella* spp. and *Leptospira* spp. since a small percentage of the sampled cattle (1.87%, 95% CI: 0.94–3.32) tested positive for *C. burnetii*. FMDV and *Brucella* spp. co-exposure was highest at 33.7% (95% CI: 30.9–36.5), followed by FMDV and *Leptospira* spp. at 21.8% (95% CI: 19.5–24.4), *Leptospira* spp. and *Brucella* spp. at 8.8% (95% CI: 7.2–10.6), FMDV and *C. burnetii* at 1.5% (95% CI: 0.7–2.8), *Brucella* spp. and *C. burnetii* at 1.0% (95% CI: 0.3–2.2), and lowest for *Leptospira* spp. and *C. burnetii* at 0.3% (95% CI: 0.0–1.2). Results of the analyses conducted using subset data for samples tested for all pathogens (*n* = 589), showed that 7.8% (95% CI: 5.7–10.2) of the cattle were seropositive to at least three pathogens. Those that were simultaneously seropositive for FMDV, *Brucella* spp., and *Leptospira* spp. were 8.4% (95% CI: 6.9–10.1), while 0.3% (95% CI: 0.0–1.2) tested positive for *Brucella* spp., *Leptospira* spp., and *C. burnetii.* Only two animals (0.3%, 95% CI: 0.0–1.2) had antibodies to all pathogens.

**Table 1:** Overall seroprevalence estimates of the various levels of co- or multiple pathogens exposure in cattle and the distribution of these estimates by zones.

Targeted pathogen outcomes	Low interface area (Zone 3)		Moderate interface area (Zone 2)		High interface area (Zone 1)		Overall seroprevalence		*χ*^2^ *p*-value
	*n*	% seropositive (95% CI)	*n*	% seropositive (95% CI)	*n*	% seropositive (95% CI)	*n*	% seropositive (95% CI)	
FMDV + *Brucella* spp.	240	21.6 (16.6–27.4)	465	28.6 (24.5–32.9)	465	44.9 (40.3–49.5)	1,170	33.7 (30.9–36.5)	<0.001
FMDV + *Leptospira* spp.	240	13.3 (9.3–18.2)	465	23.6 (19.8–27.7)	465	24.3 (20.4–28.4)	1,170	21.8 (19.5–24.3)	0.002
*Leptospira* spp. + *Brucella* spp.	240	5.8 (3.2–9.5)	465	8.3 (6.0–11.2)	465	10.7 (8.0–13.9)	1,170	8.8 (7.2–10.6)	0.085
FMDV + *Brucella* spp. + *Leptospira* spp.	240	5.0 (2.6–8.5)	465	7.7 (5.4–10.5)	465	10.5 (8.0–13.9)	1,170	8.4 (6.9–10.1)	0.027
FMDV + *C. burnetii*	121	2.4 (0.5–7.0)	239	0.8 (0.1–2.9)	229	1.7 (0.4–4.4)	589	1.5 (0.7–2.8)	0.459
*Brucella* spp. + *C. burnetii*	121	0.8 (0.0–4.5)	239	0.4 (0.0–2.3)	229	1.7 (0.4–4.4)	589	1.0 (0.3–2.2)	0.350
*Leptospira* spp. + *C. burnetii*	121	0.8 (0.0–4.5)	239	0.0 (0.0–1.5)	229	0.4 (0.0–2.4)	589	0.3 (0.0–1.2)	0.422
*Brucella* spp. + *Leptospira* spp. + *C. burnetii*	121	0.8 (0.0–4.5)	239	0.0 (0.0–1.5)	229	0.4 (0.0–2.4)	589	0.3 (0.0–1.2)	0.422
Exposure of cattle to at least one pathogen	121	74.4 (65.6–81.9)	239	88.7 (84.0–92.4)	229	94.3 (90.5–96.9)	589	88.0 (85.0–90.5)	<0.001
Exposure of cattle to at least two pathogens	121	28.9 (21.0–37.8)	239	41.8 (35.5–48.3)	229	48.4 (41.8–55.1)	589	41.7 (37.7–45.8)	0.002
Exposure of cattle to at least three pathogens	121	4.9 (1.8–10.4)	239	6.2 (3.5–10.1)	229	10.9 (7.1–15.6)	589	7.8 (5.7–10.2)	0.074
Exposure of cattle to all pathogens	121	0.8 (0.0–4.5)	239	0.0 (0.0–1.5)	229	0.4 (0.0–2.4)	589	0.3 (0.0–1.2)	0.422

n, number of cattle tested; CI, confidence interval.

The co-exposure of FMDV and *Brucella* spp. differed significantly by zones (*p* < 0.001). Highest co-exposure was found among cattle raised in zone 1 (high interface area) than those in zone 3 (Table 1). The co-exposure of cattle to FMDV and *Leptospira* spp.; simultaneous exposure to FMDV, *Brucella* spp. and *Leptospira* spp.; and to at least one, and two pathogens also varied statistically by zones and were all higher in zone 1 than the other zones. The other co- or multiple pathogen exposures did not vary significantly by zones (Table 1).

### 3.2 Risk factors associated with the co-exposures of the targeted pathogens

#### 3.2.1 Univariable results

Univariate and multivariate analyses did not yield many significant factors compared to the univariable and multivariable Bayesian models, respectively. Consequently, we did not report these results. From the univariable Bayesian models, the co-exposure of cattle to FMDV and *Leptospira* spp. was significantly associated with animal sex (female), raising of cattle in moderate (zone 2) and high interface areas (zone 1); pastoral herd management practice, animals sharing a common grazing reserve, grazing of animals in the MMNR and on pastures shared between villages as well as a positive history of abortions in sampled herds (Table 2). The factors that predicted FMDV and *Brucella* spp. co-exposure included animals’ sex (female), zones (moderate and high interface areas), pastoral herd management practice, grazing of animals on pastures shared within and between villages; and in the MMNR, contact of animals with others from a different herd during grazing, utilization of watering points shared between villages and mixing of cattle with others from a different herd at watering points. For *Leptospira* spp. and *Brucella* spp. co-exposure, animal sex (female), zones (high interface area), pastoral herd management practice, grazing of cattle in pastures shared between villages and in the MMNR; and also contact of animals with others from a different herd during grazing were all identified as significant predictors of cattle co-exposure to these two pathogens. The co-exposure of *Brucella* spp. and *C. burnetii* in cattle was positively associated with pastoral herd management practice, grazing of animals on pastures shared within and between villages; contact of animals with others from a different herd during grazing, sharing of watering points within and between villages; and mixing of animals with others from a different herd at watering points. FMDV and *C. burnetii* co-exposure was significantly associated with grazing of animals on pastures shared between villages. Lastly, the significant factors found to be associated with the concurrent exposure of cattle to FMDV, *Brucella* spp. and *Leptospira* spp. were animal sex (female), zones (high interface area), pastoral herd management practice, and grazing of animals on pastures shared within villages and in the MMNR. Cattle herd size included in the models as a log-transformed or categorical factor was not significantly associated with any of the outcomes of interest and was not considered in further analyses.

**Table 2:** Univariable Bayesian mixed model results showing factors that were significantly associated with at least one of the considered outcomes.

Variables and categories	FMDV + *Leptospira* spp.			FMDV + *Brucella* spp.			*Leptospira* spp. + *Brucella* spp.			*Brucella* spp. + *C. burnetii*			FMDV *+ C. burnetii*			FMDV + *Brucella* spp. + *Leptospira* spp.		
	Mean* (SE)	l-95%	u-95%	Mean (SE)	l-95%	u-95%	Mean (SE)	l-95%	u-95%	Mean (SE)	l-95%	u-95%	Mean (SE)	l-95%	u-95%	Mean (SE)	l-95%	u-95%
**Sex**																		
Male	(Ref.)			(Ref.)			(Ref.)			(Ref.)			(Ref.)			(Ref.)		
Female	0.70	**0.30**	**1.13**	1.00	**0.61**	**1.41**	1.20	**0.52**	**1.97**	−0.47	−2.24	1.48	−0.55	−2.06	1.03	1.06	**0.37**	**1.81**
Zones**																		
Zone 3	(Ref.)			(Ref.)			(Ref.)			(Ref.)			(Ref.)			(Ref.)		
Zone 2	0.74	**0.30**	**1.22**	0.41	−0.02	0.87	0.41	−0.23	1.10	−0.61	−3.97	2.77	−1.19	−3.34	0.74	0.52	−0.20	1.30
Zone 1	0.78	**0.33**	**1.27**	1.22	**0.78**	**1.68**	0.70	**0.05**	**1.39**	1.11	−1.08	4.17	−0.34	−2.12	1.39	0.91	**0.22**	**1.68**
**Herd management practice**																		
Sedentary	(Ref.)			(Ref.)			(Ref.)			(Ref.)			(Ref.)			(Ref.)		
Pastoral	0.49	**0.18**	**0.81**	0.84	**0.52**	**1.17**	0.58	**0.17**	**1.01**	2.10	0.06	5.05	0.51	−0.93	1.96	0.68	**0.23**	**1.16**
**Shared a common grazing reserve**																		
No	(Ref.)			(Ref.)			(Ref.)			(Ref.)			(Ref.)			(Ref.)		
Yes	0.68	**0.36**	**1.02**	0.29	−0.07	0.67	0.34	−0.12	0.78	1.77	−0.05	3.79	0.79	−0.72	2.20	0.49	−0.01	0.99
**Grazed animals on pastures shared within village**																		
No	(Ref.)			(Ref.)			(Ref.)			(Ref.)			(Ref.)			(Ref.)		
Yes	0.41	−0.08	0.94	0.92	**0.38**	**1.47**	0.83	**0.08**	**1.68**	30.40	**0.40**	**160.09**	0.55	−1.58	3.68	0.96	**0.11**	**1.96**
**Grazed animals on pastures shared between villages**																		
No	(Ref.)			(Ref.)			(Ref.)			(Ref.)			(Ref.)			(Ref.)		
Yes	0.54	**0.21**	**0.87**	0.37	**0.02**	**0.75**	0.25	−0.22	0.74	1.84	**0.04**	**3.91**	0.83	−0.73	2.32	0.40	−0.09	0.90
**Grazed cattle in the MMNR**																		
No	(Ref.)			(Ref.)			(Ref.)			(Ref.)			(Ref.)			(Ref.)		
Yes	0.44	**0.12**	**0.77**	0.74	**0.42**	**1.05**	0.51	**0.11**	**0.92**	1.05	−0.71	3.15	0.01	−1.50	1.47	0.61	**0.14**	**1.09**
**Contact with other cattle from a different herd during grazing**																		
No	(Ref.)			(Ref.)			(Ref.)			(Ref.)			(Ref.)			(Ref.)		
Yes	0.35	−0.12	0.83	0.53	**0.07**	**0.99**	0.73	**0.05**	**1.51**	21.27	**0.38**	**96.69**	−0.95	−2.50	0.71	0.69	−0.04	1.50
**Shared watering points within village**																		
No	(Ref.)			(Ref.)			(Ref)			(Ref)			(Ref)			(Ref)		
Yes	0.63	−0.30	1.65	0.96	−0.02	2.02	0.40	−0.77	1.87	68.24	**0.15**	**299.52**	64.49	**0.86**	**299.93**	0.89	−0.56	2.87
**Shared watering points between villages**																		
No	(Ref.)			(Ref.)			(Ref.)			(Ref.)			(Ref.)			(Ref.)		
Yes	0.63	−0.30	1.65	0.68	0.36	1.02	0.32	−0.14	0.77	10.08	**0.97**	**39.06**	0.49	−0.91	2.03	0.38	−0.09	0.80
**Mixed cattle with others from a different herd at watering points**																		
No	(Ref.)			(Ref.)			(Ref.)			(Ref.)			(Ref.)			(Ref.)		
Yes	0.38	−0.00	0.79	0.44	**0.06**	**0.84**	0.37	−0.16	0.94	23.03	**0.60**	**216.58**	−0.29	−1.74	1.33	0.39	−0.16	0.99
**Bought livestock in previous year**																		
No	(Ref.)			(Ref.)			(Ref.)			(Ref.)			(Ref.)			(Ref.)		
Yes	−0.05	−0.41	0.31	0.10	−0.26	0.48	−0.06	−0.54	0.43	0.97	−1.26	3.88	1.44	−0.55	4.37	−0.09	−0.61	0.42
**History of abortions in sampled**																		
No	(Ref.)			(Ref.)			(Ref.)			(Ref.)			(Ref.)			(Ref.)		
Yes	0.41	**0.08**	**0.88**	0.22	−0.10	0.54	0.07	−0.34	0.49	−0.03	−1.79	1.72	−0.80	−2.46	0.65	0.20	−0.25	0.64

^*^Mean of posterior distribution; SE, standard deviation of the posterior distributions; Ref., Reference category; l-95% and u-95% represents the lower and upper Bayesian credible intervals, respectively. MMNR, Maasai Mara National Reserve. The bolded Bayesian credible intervals are for variables that were statistically significant. ^**^Zone 3, low interface area; Zone 2, moderate interface area; zone 1, high interface area.

#### 3.2.2 Multivariable results

The multivariable Bayesian results are summarized in Table 3. From these results, the co-exposure of cattle to FMDV and *Leptospira* spp. was associated with animal sex, with more female cattle being seropositive than males. Cattle raised in moderate (zone 2) and high interface areas (zone 1) also had a higher probability of co-exposure to these pathogens than those in low interface areas. FMDV and *Leptospira* spp. co-exposure was also positively associated with pastoral husbandry practices and animals sharing a common grazing reserve. For FMD and *Brucella* spp.; and *Leptospira* spp. and *Brucella* spp. co-exposures, and also for animals with simultaneous exposure to FMDV, *Brucella* spp. and *Leptospira* spp., female cattle were also likely to test seropositive than males. Raising animals in high interface areas (zone 1) was also identified as an important predictor for these outcomes.

**Table 3:** Results of the multivariable Bayesian mixed models showing variables found to be significantly associated with the considered outcomes.

Targeted pathogens co-exposures	Variables and categories	Mean^*^	SE	l-95% CI	u-95% CI	Rhat	Bulk ESS	Tail ESS	^a^Variance	VPC
FMDV and *Leptospira* spp.	Animal sex								0.16	0.08
	Male	(Ref.)								
	Female	0.81	0.21	0.40	1.21	1.00	6,891	3,260		
	Zones									
	Zone 3	(Ref.)								
	Zone 2	0.58	0.25	0.11	1.07	1.00	4,291	2,795		
	Zone 1	0.02	0.38	−0.72	0.76	1.00	3,359	2,670		
	Herd management practice									
	Sedentary	(Ref.)								
	Pastoral	0.57	0.28	0.00	1.14	1.00	4,047	2,648		
	Shared a common grazing reserve									
	No	(Ref.)								
	Yes	0.60	0.19	0.22	0.99	1.00	5,585	3,210		
FMDV and *Brucella* spp.	Animal sex								0.21	0.15
	Male	(Ref.)								
	Female	1.16	0.21	0.77	1.58	1.00	6,771	3,124		
	Zones									
	Zone 3	(Ref.)								
	Zone 2	0.48	0.25	−0.00	0.96	1.00	5,396	3,118		
	Zone 1	1.45	0.25	0.96	1.97	1.00	4,334	3,360		
*Leptospira* spp. and *Brucella* spp.	Animal sex								0.07	0.30
	Male	(Ref.)								
	Female	1.30	0.38	0.62	2.08	1.00	4,929	2,566		
	Zones									
	Zone 3	(Ref.)								
	Zone 2	0.47	0.35	−0.19	1.17	1.00	3,328	2,839		
	Zone 1	0.86	0.34	0.23	1.54	1.00	3,259	2,428		
*Brucella* spp. and *C. burnetii*	Grazed of animals on pastures shared within villages								_	_
	No	(Ref.)								
	Yes	666.64	984.01	3.78	3681.01	1.04	94	111		
	Contact with other cattle from a different herd during grazing									
	No	(Ref.)								
	Yes	566.58	769.45	0.12	2807.61	1.02	85	112		
	Shared watering points between villages.									
	No	(Ref.)								
	Yes	248.37	434.91	258	1397.81	1.04	82	105		
	Mixed cattle with others from a different herd at watering points									
	No	(Ref.)								
	Yes	289.83	469.09	1.73	1760.70	1.06	68	73		
FMDV and *C. burnetii*	Shared watering points within village								0.02	0.28
	No	(Ref.)								
	Yes	75.45	102.36	0.93	372.52	1.00	895	490		
	Source of breeding bull									
	Own bull	(Ref.)								
	Bull from another farm	1.51	0.77	0.00	3.02	1.00	6,663	2,726		
FMDV, *Brucella* spp. and *Leptospira* spp.									0.07	0.48
	Animal sex									
	Male	(Ref.)								
	Female	1.18	0.37	0.51	1.95	1.00	5,729	2,910		
	Zones									
	Zone 3	(Ref.)								
	Zone 2	0.57	0.39	−0.17	1.37	1.00	3,840	2,370		
	Zone 1	1.06	0.39	0.35	1.88	1.00	3,758	2,508		

^*^Mean of the posterior distribution; ^a^Variance of the varying effect (herd ID); VPC, variance partition coefficient; SE, standard error of the posterior distribution; Ref, reference category; l-95% CI and u-95% CI denote the lower and upper Bayesian credible intervals, respectively. Bulk and Tail ESS are effective sample size measures. The variance and VPC for *Brucella* spp. and *C. burnetii* co-exposure final model (both marked with dashes) were not computed as some parts of the model did not converge.

In the analyses of factors associated with the concurrent exposure of cattle to FMDV, *Brucella* spp. and *Leptospira* spp., alternative final multivariable Bayesian models comprising various pairs of significant covariates from the univariable models (i.e., animal sex and herd management practice; animal sex and zones; animal sex and animals sharing a common grazing reserve; animal sex and grazing of cattle in the MMNR) could be fitted to the data. However, a final model with animal sex and zones as the fixed effects was selected over the others as a proxy to explain some predictors in the alternative final models mentioned above. In this case, zone 1 was used to explain pastoral production system and grazing of cattle in the MMNR, predominant livestock husbandry practices in this area. The significant predictors of *Brucella* spp. and *C. burnetii* co-exposure were grazing of animals on pastures shared within villages, contact of cattle with others from a different herd during grazing, mixing of cattle with others from a different herd at watering points and sharing of watering points between villages. For FMDV and *C. burnetii* co-exposure, sharing of watering points within villages and use of a breeding bull from another farm were revealed as significant factors associated with cattle seropositivity to these pathogens.

The results from the model fit diagnostics revealed that the Rhat values were 1.0 for five of the six final models (Table 3), a confirmation that the algorithms for these models reached stationary distributions and converged. Slightly large Rhat values, though not exceeding the recommended limit of 1.1, were obtained from the final model for *Brucella* spp. and *C. burnetii* co-exposure, showing that some parts of this model failed to converge. The gradual increase of the number of iterations up to 50,000 and the *adapt*_*delta* (within the *brms* package) up to 0.999 to minimize divergent transitions that affect the validity of posterior draws, all did not improve the results. The estimated VPCs for the converged final models together with their corresponding random effect variances for the posterior predicted distributions are presented in Table 3. For these models, the fairly symmetrical histograms of the posterior samples and the trace plots of the standard deviation for the residuals, intercept and slope of the fixed effects, with a random scatter above and below the mean values also depicted convergence of the algorithms (Supplementary Figures S1–S5). Furthermore, the posterior predictive checks for all the final models demonstrated similar density plots for observed (*y*) and predicted data (*y rep*), implying fit of the models to the data (Supplementary Figures S6–S11). Comparison of the competing (null, global and final) Bayesian models showed that all the final models had better fit of the data as they had the lowest leave-one-out information criteria (LOOIC) and WAIC values.

## 4 Discussion

This study confirmed cattle’s concomitant exposure to the targeted pathogens in the wildlife-livestock overlap areas of Narok County and revealed that a large percentage of these hosts (41.7%) were seropositive for at least two of the investigated pathogens. This finding aligns with results of other studies elsewhere that have shown mixed infections and/or co-exposures as a common phenomenon among livestock animals raised in different production systems (35–37), since these hosts could be continually exposed to heterogeneous pathogens present in their habitats. Except for FMDV, vaccination of cattle against the other targeted pathogens has not been adopted in the area hence the observed co-exposures are associated with natural infections with these pathogens.

As depicted by the computed VPCs, most co-exposures were either moderately or highly correlated among cattle within herds (VPCs range: 0.15–0.48) except for *Brucella* spp. and *C. burnetii* co-exposure that was not calculated and FMDV and *Leptospira* spp. co-exposure that was considerably low (0.08). This finding illustrates a considerable degree of transmission of the investigated pathogens within herds in the area. The targeted pathogens are highly contagious and share transmission routes thus within herd contact rates between sick and healthy cattle could lead to a substantial number of animals being infected.

The highest co-exposures were recorded between FMDV, *Brucella* spp. and *Leptospira* spp. which is attributable to the high animal-level seroprevalence estimates reported for these pathogens among cattle in the area (9, 13, 15), hence a high likelihood of concurrent exposures in the same animal. For *C. burnetii*, the estimated seroprevalence (1.87%) was very low. There are limited published studies on this pathogen among livestock populations in the area, but this value aligned with the seroprevalence estimates documented by other studies implemented within Kenya in areas with comparable livestock production systems to MME (38–40). Those studies reported significantly lower seropositivity estimates for *C. burnetii* among cattle relative to small ruminants (sheep and goats), although the latter were not sampled in our study. Even so, a recent systematic review also documented different seroprevalence estimates of *C. burnetii* among livestock species across Africa (41), presumably due to varied epidemiological drivers in these contexts or the use of serological tests with different specificity and sensitivity estimates.

The specific impacts of multiple infections and/or exposures of cattle with the investigated pathogens remain poorly understood, but serious animal health consequences ascribed to the synergistic effects of these pathogens could be manifested among infected cattle. This, for instance, could include high morbidity and death rates; severe illness, increased animal susceptibility to secondary pathogens due to immune depression and clinical misdiagnosis as all manifest non-specific syndromes (37). Cattle infections with the focused pathogens cause significant reproductive disorders. In mixed infections, infected animals could manifest worse health outcomes leading to serious economic losses. Such economic losses can be compounded by trade restrictions of livestock and their products during FMDV outbreak (23). These pathogens also cause a significant decline in milk yields among infected animals which could affect the food security of the local farmers. The detection of the three zoonotic pathogens in cattle is also a major food safety concern to the local communities as they can get exposed through the consumption of raw animal products including milk and meat. Livestock keepers, herders and individuals in the livestock value chain including those working in abattoirs in these locations are also at risk of infections with multiple zoonotic pathogens. While we could not confirm humans’ exposure to the targeted zoonotic agents as they were not sampled, brucellosis has been reported among these hosts in the area (15).

FMDV and *Brucella* spp., *Leptospira* spp. and *Brucella* spp., co-exposures and the simultaneous exposure of cattle to FMDV, *Brucella* spp., and *Leptospira* spp. were all recorded highest among animals sampled in zone 1 compared to those from zones 2 and 3. These findings may be due to grazing of cattle in wildlife inhabited locations, particularly, the MMNR and surrounding conservancies. Indeed, grazing of cattle in MMNR was significantly associated with the above outcomes. The investigated pathogens infect a great diversity of wildlife including the African buffalo (*Syncerus caffer*), notable self-sustaining reservoir hosts found in the area that could inadvertently transmit these pathogens to livestock through “spill over” (42–45). Even though wildlife were not screened for the focal pathogens, earlier studies conducted in the area revealed exposure of various wildlife species including the African buffalo to *Brucella* spp., *C. burnetii* among other zoonotic pathogens (46) and FMDV (47), further supporting the above hypothesis. The different land use types embraced by farmers in the area could also account for the variations in co-exposures by zones. Semi-nomadic pastoral system is the primary livestock grazing strategy used in zone 1 and was also identified as an important predictor of the above co- or multiple pathogen exposures. This livestock production system permit close interactions of animals within and between herds as they share pasture or congregate at watering points which could promote the transmission levels of infectious agents between hosts. Livestock movement that is characteristic of this system could also increase the spread of infectious pathogens via environmental contamination, further enhancing the indirect transmission pathways of the targeted pathogens. FMDV and *Leptospira* spp. co-exposure was significantly higher among cattle raised in zones 1 and 2 compared to zone 3, but not between those in zones 1 and 2. This finding suggest that cattle co-exposure to these two pathogens could have been influenced by several other factors further to the effects of land use differences by zones evaluated in this study. Other epidemiological studies should identify the ecological drivers of these two pathogens in the area.

The sharing of common grazing reserves among cattle and watering points within villages were identified as significant predictors of FMDV and *Leptospira* spp., and FMDV and *C. burnetii* co-exposures, respectively. The co-exposure of cattle to *Brucella* spp. and *C. burnetii* was also significantly associated with grazing of animals on pastures shared within villages, animals sharing watering points between villages and mixing of cattle with others of a different herd at watering points. Given that the targeted pathogens are excreted by infected animals in urine and feces among other secretions (17, 19, 48, 49), all these findings are related to environmental contamination which could act as a long term source of infections for animals with shared husbandry practices owing the long persistence nature of these pathogens in the environment, a period estimated to last for weeks to months or even years and strongly influenced by climatic, geographical and edaphic factors including temperature, humidity, precipitation, soil moisture, ultraviolet light, pH and salinity (50–55).

The association between the use a breeding bull from another farm and cattle co-exposure to FMDV and *C. burnetii* could be because both pathogens are excreted in semen in large quantities (22, 53) which could facilitate their transmission to naïve cattle within herds via sexual contact. Also, *Brucella* spp. and *C. burnetii* co-exposure was predicted by cattle contacts with others from a different during grazing which could promote the transmission of these pathogens between infected and susceptible hosts as earlier explained.

This study also showed that female animals were more co-exposed to FMDV and *Leptospira* spp., FMDV and *Brucella* spp., and *Leptospira* spp. and *Brucella* spp. than males as with the case of the concurrent exposure of cattle to FMDV, *Brucella* spp. and *Leptospira* spp. This finding might be due to continual infections of females with the above pathogens since compared to males, these animals are kept by livestock producers over many years as a source of nutrition, income and breeding purposes.

There were also several limitations in our study. For instance, cattle co-exposures to the targeted pathogens were determined by screening for the presence of specific immunoglobulins against these pathogens that remain detectable from months to years following infection (56–59), thus we could not ascertain if these outcomes were as a result of concurrent, secondary simultaneous or consecutive infections or determine when cattle got exposed. Cattles’ exposure to FMDV was also determined using anti-NSP tests that are limited since non-detectable immunoglobulins against NSP antibodies could be induced among previously vaccinated and later infected cattle having little or without systemic infection (60), potentially leading to false negative reactions. The currently used FMDV vaccine in Kenya are purified (61), but the use of non-purified FMDV vaccines could also induce anti-NSPS (62), thus affecting animals’ exposure interference based on these results. Cattle were also exclusively tested for antibodies against *Brucella* spp., *Leptospira* spp. and *C. burnetii* using commercial ELISA kits instead of bacterial culture, microscopic agglutination test (MAT) and complement fixation test (CFT), gold standard tests for these pathogens, respectively (63–65). Nonetheless, these reference tests are less sensitive compared to ELISA tests (19, 66, 67). Samples were also not tested for anti-*Brucella* spp. using the Rose Bengal test (RBT) because of logistical constraints. In spite of this, indirect ELISA tests have been found to be more sensitive than RBT in pastoral areas (25, 68). Additionally, immunological cross-reactions between *C. burnetii* and other Gram negative bacteria including *Legionella* spp., *Bartonella* spp. and *Chlamydia* spp. is also recognized (69, 70). Similarly, *Salmonella* spp., *Campylobacter* spp., *Francisella tularensis*, *Yersinia enterocolitica* 0:9, *Pasteurella* spp. and *Escherichia coli* O116 and O157, all cross-react with *Brucella* spp. due to antigenically related lipopolysaccharide epitopes (71). This could have led to false positivity and slight over-estimation of the co-exposures. We also sampled cattle in confluent zones which could have affected the estimated co-exposures by zones since cattle could have moved between these sites.

## 5 Conclusion

Our study provides immunological evidence of cattle co-exposure with the investigated pathogens. We observed highest co-exposures between FMDV, *Brucella* spp. and *Leptospira* spp. Based on study locations, more cattle with FMDV and *Brucella* spp., and *Brucella* spp. and *Leptospira* spp. co-exposures and those concurrently exposed to FMDV, *Brucella* spp. and *Leptospira* spp. were recorded in zone 1 compared to zones 2 and 3. In contrast, FMDV and *Leptospira* spp. co-exposure was higher among cattle raised in both zones 1 and 2 than zone 3. Since some of the targeted pathogens are zoonotic and have been previously detected in wildlife and humans as earlier discussed, there is a need to develop an integrated One Health biosurveillance control intervention for these pathogens rather than focusing on single pathogens to reduce their transmission and morbidities in livestock and risks of “spillover” to humans. Vaccination of livestock against the targeted zoonotic pathogens should also be considered because these hosts are the critical sources of human infections. While a previous study showed that FMDV significantly affect livestock-sourced livelihoods in the area (3), we recommend further studies to understand the incidence and economic impacts of *Brucella* spp., *Leptospira* spp. and *C. burnetii* in livestock and human populations.

## Data availability statement

The original contributions presented in the study are included in the article/Supplementary material, further inquiries can be directed to the corresponding author.

## Ethics statement

The animal studies were approved by the Animal Care and Use Committee of the International Livestock Research Institute (ILRI) (reference number: ILRI-IACUC 2016-20), while ethical approval was granted by the ILRI's Institutional Research Ethics Committee (reference number: ILRI-IREC 2016-02). The studies were conducted in accordance with the local legislation and institutional requirements. Written informed consent was obtained from the owners for the participation of their animals in this study.

## Author contributions

SM: Conceptualization, Formal analysis, Investigation, Methodology, Writing – original draft, Writing – review & editing. DN: Conceptualization, Data curation, Formal analysis, Investigation, Methodology, Project administration, Resources, Software, Supervision, Validation, Visualization, Writing – original draft, Writing – review & editing. HL: Conceptualization, Investigation, Methodology, Supervision, Writing – review & editing. MM: Investigation, Writing – review & editing. RN: Investigation, Methodology, Writing – review & editing. AM: Investigation, Writing – review & editing. GW: Investigation, Methodology, Writing – review & editing. JA: Investigation, Writing – review & editing. BB: Conceptualization, Funding acquisition, Investigation, Methodology, Project administration, Resources, Supervision, Writing – review & editing.

## Supplementary material

Supplementary Figure S1 Histogram of the posterior samples and trace plots from the final model for FMDV and *Brucella* spp. co-exposure.

Supplementary Figure S2 Histogram of the posterior samples and trace plots from the final model for FMDV and *Leptospira* co-expousure.

Supplementary Figure S3 Histogram of the posterior samples and trace plots from the final model for *Brucella* spp. and *Leptospira* spp. co-exposure.

Supplementary Figure S4 Histogram of the posterior samples and trace plots from the final model for FMDV, *Brucella* spp. and *Leptopira* spp. concurrent exposure.

Supplementary Figure S5 Histogram of the posterior samples and trace plots from the final model for FMDV and *C. burnetii* co-exposure.

Supplementary Figure S6 Density plot from the final model for FMDV and *Brucella* spp. co-exposure.

Supplementary Figure S7 Density plot from the final model for FMDV and *Leptospira* spp. co-exposure.

Supplementary Figure S8 Density plot from the final model for *Brucella* spp. and *Leptospira* spp. co-exposure.

Supplementary Figure S9 Density plot from the final model for FMDV, *Brucella* spp. and *Leptospira* spp. concurrent exposure.

Supplementary Figure S10 Density plot from the final model for FMDV and *C. burnetii* co-exposure.

Supplementary Figure S11 Density plot from the final model for *Brucella* spp. and *C. burnetii* co-exposure.
