# Supplementary figures and images for: Multiple pathogens co-exposure and associated risk factors among cattle reared in a wildlife-livestock interface area in Kenya
Source: Front Vet Sci. 2024 Jul 25;11:1415423. doi: 10.3389/fvets.2024.1415423 (PMC11306132; doi:10.3389/fvets.2024.1415423)

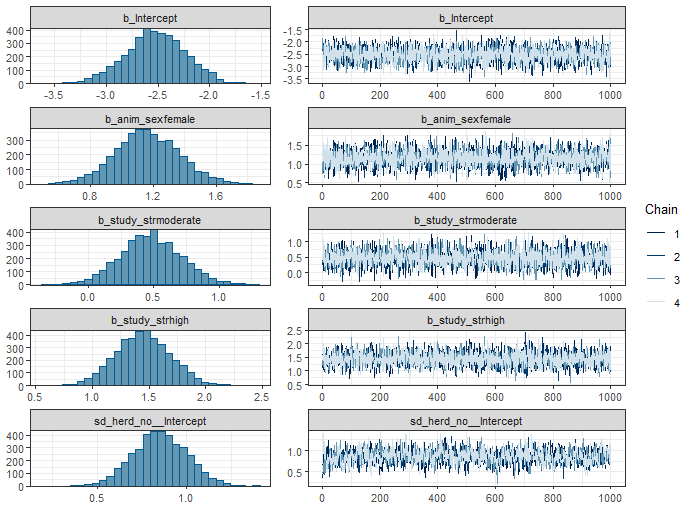

Supplement: Supplementary Figure S1 — Histogram of the posterior samples and trace plots from the final model for FMDV and Brucella spp. co-exposure. [file Image_1.TIFF]

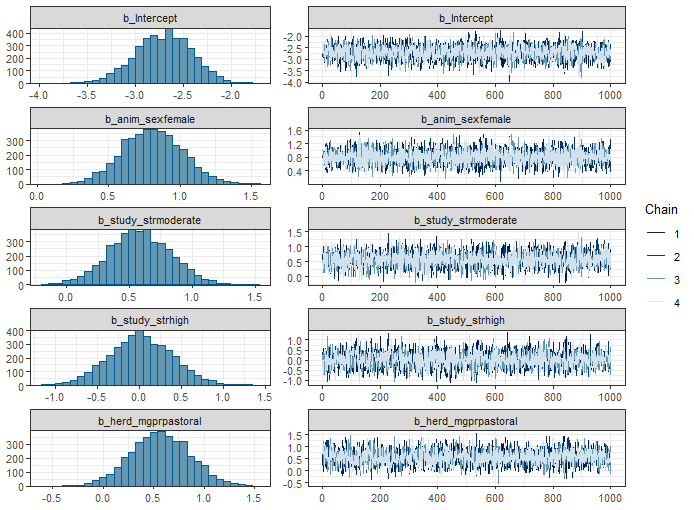

Supplement: Supplementary Figure S2 — Histogram of the posterior samples and trace plots from the final model for FMDV and Leptospira co-expousure. [file Image_2.TIFF]

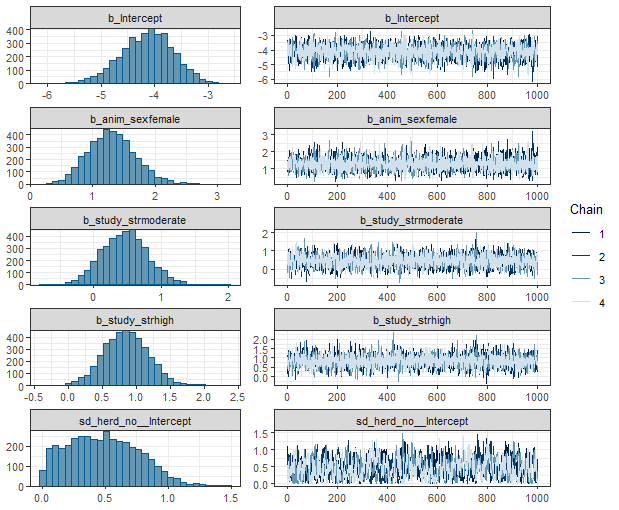

Supplement: Supplementary Figure S3 — Histogram of the posterior samples and trace plots from the final model for Brucella spp. and Leptospira spp. co-exposure. [file Image_3.TIFF]

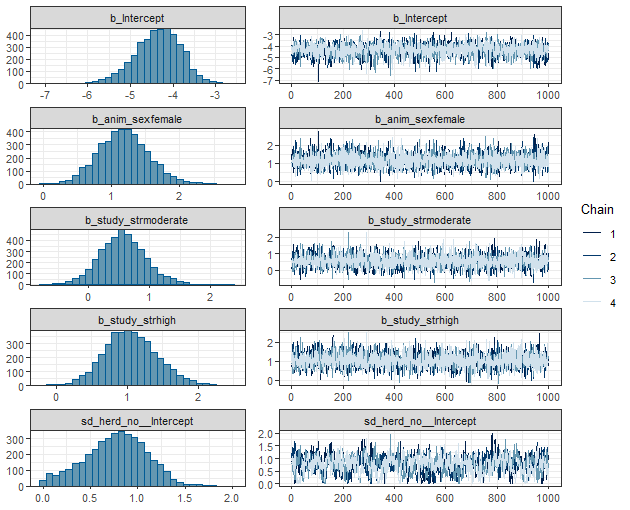

Supplement: Supplementary Figure S4 — Histogram of the posterior samples and trace plots from the final model for FMDV, Brucella spp. and Leptopira spp. concurrent exposure. [file Image_4.TIFF]

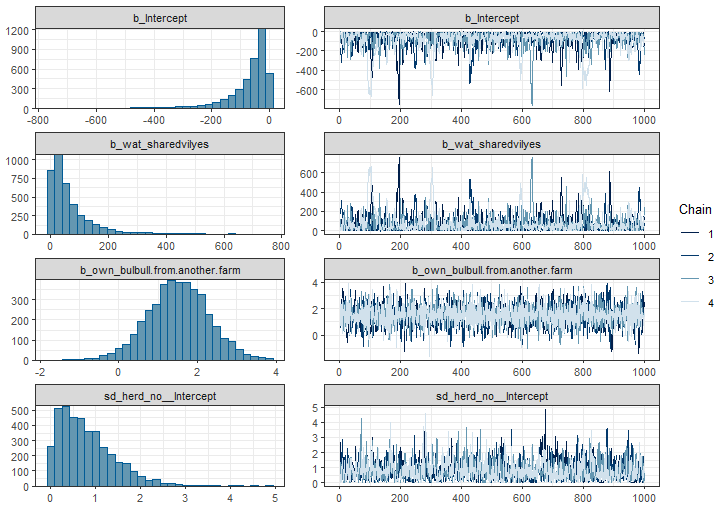

Supplement: Supplementary Figure S5 — Histogram of the posterior samples and trace plots from the final model for FMDV and C. burnetii co-exposure. [file Image_5.TIFF]

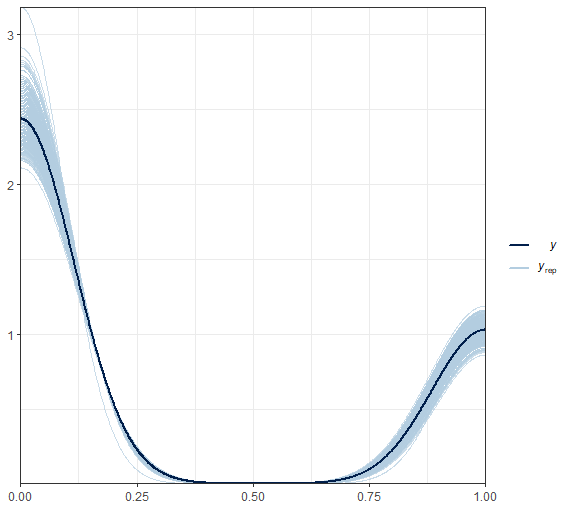

Supplement: Supplementary Figure S6 — Density plot from the final model for FMDV and Brucella spp. co-exposure. [file Image_6.TIFF]

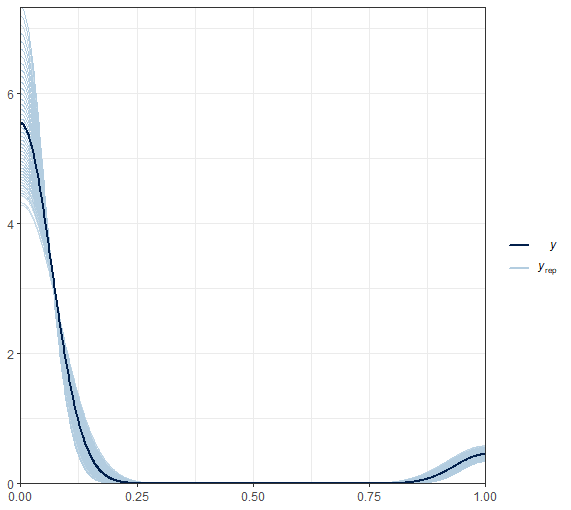

Supplement: Supplementary Figure S7 — Density plot from the final model for FMDV and Leptospira spp. co-exposure. [file Image_7.TIFF]

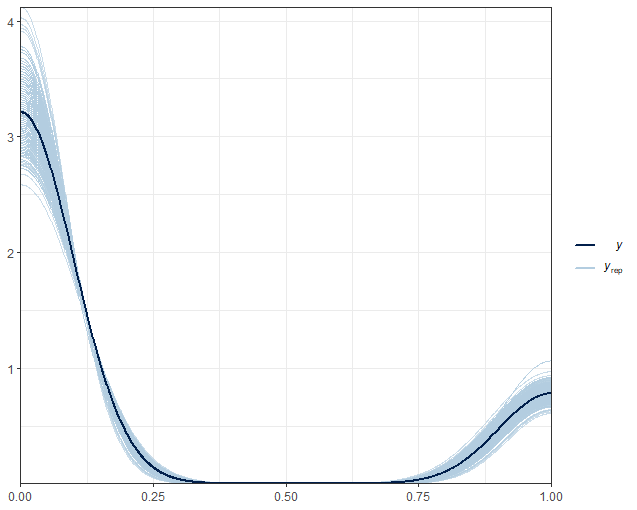

Supplement: Supplementary Figure S8 — Density plot from the final model for Brucella spp. and Leptospira spp. co-exposure. [file Image_8.TIFF]

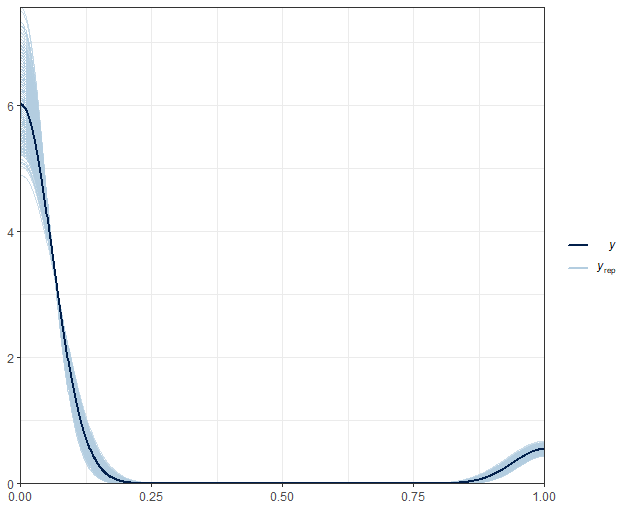

Supplement: Supplementary Figure S9 — Density plot from the final model for FMDV, Brucella spp. and Leptospira spp. concurrent exposure. [file Image_9.TIFF]

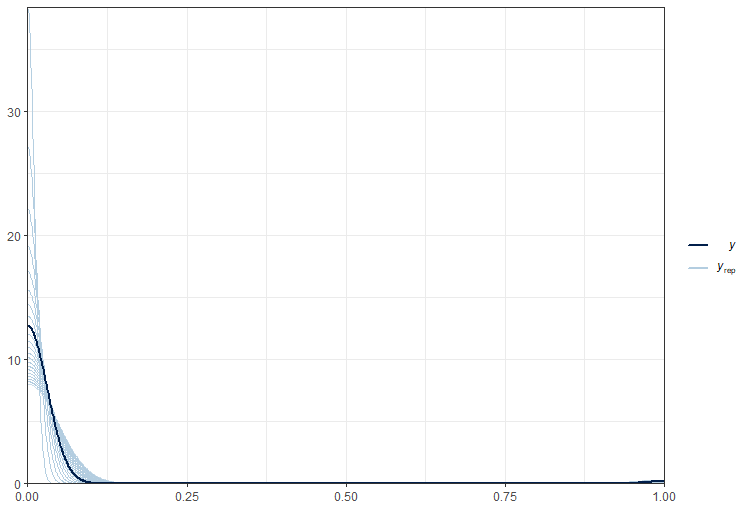

Supplement: Supplementary Figure S10 — Density plot from the final model for FMDV and C. burnetii co-exposure. [file Image_10.TIFF]

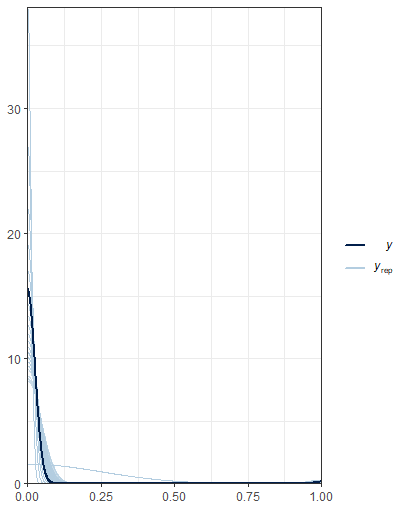

Supplement: Supplementary Figure S11 — Density plot from the final model for Brucella spp. and C. burnetii co-exposure. [file Image_11.TIFF]
